# Reliability of heart rate in reflecting cardiac sympathetic overdrive in type 2 diabetes mellitus

**DOI:** 10.1007/s10286-024-01054-z

**Published:** 2024-07-22

**Authors:** Raffaella Dell’Oro, Fosca Quarti-Trevano, Stefano Ciardullo, Gianluca Perseghin, Giuseppe Mancia, Guido Grassi

**Affiliations:** 1 Department of Medicine, Clinica Medica, Surgery University Milano-Bicocca, Via Pergolesi 33, 20052 Monza, Milan Italy; 2 Department of Medicine and Rehabilitation, Department of Medicine and Surgery, Policlinico Dii Monza, University Milano-Bicocca, Monza, Milan Italy; 3 University Milano-Bicocca, Monza, Milan Italy

**Keywords:** Type 2 diabetes, Heart rate, Sympathetic activity, Cardiovascular risk, Sympathetic nerve traffic, Plasma norepinephrine

## Abstract

**Purpose:**

Clinical trials have shown that in type 2 diabetes mellitus (T2D) resting office heart rate (HR) values > 70 beats/minute are associated with an increased cardiovascular risk, a worse prognosis and an unfavorable outcome. The present study was aimed at investigating whether the above mentioned treshold HR values reflect a sympathetic overdrive of marked degree.

**Methods:**

In 58 T2D patients (age range: 39–57 years) without signs of autonomic neuropathy and in 52 age-matched healthy controls, we assessed muscle sympathetic nerve activity (MSNA, microneurography) and venous plasma norepinephrine (NE, HPLC), subdividing the study population in different subgroups according to their clinic and 24-h HR values.

**Results:**

In T2D progressively greater clinic and 24-h HR values were accompanied by progressive increases in MSNA and NE. HR cutoff values indicated by clinical trials as associated with an increased cardiovascular risk (> 70 beats/minute) were accompanied by MSNA values significantly higher than those detected in patients with lower HR, this being the case also for NE. In T2D both MSNA and NE were significantly related to clinic (*r* = 0.93, *P* < 0.0001 and *r* = 0.87, *P* < 0.0001, respectively) and 24-h (*r* = 0.92, *P* < 0.0001 and *r* = 0.84, *P* < 0.0001, respectively) HR. The MSNA and NE behaviour observed in T2D was not detected in healthy controls.

**Conclusions:**

In T2D clinic HR values allow to detect patients with a greater sympathetic overactivity. Considering the adverse clinical impact of the sympathetic overdrive on prognosis, our data emphasize the need of future studies investigating the potential usefulness of lifestyle and pharmacological interventions exerting sympathomodulatory effects.

## Introduction

In type 2 diabetes mellitus (T2D) elevated resting heart rate (HR) values are associated with an increased cardiovascular risk independently on other confounders. This has been shown years ago by the results of prospective longitudinal studies [1, 2] and more recently in interventional clinical trials, in which the hazard ratios for cardiovascular death, hospitalization for heart failure as well as all cause death were higher in patients with T2D showing resting HR values greater than 70–75 beats/minute than in those displaying lower values [3, 4]. Adjustments for other concomitant risk factors did not modify the studies conclusions, which were very recently confirmed by a subanalysis of the data collected in a large scale prospective study [5]. Indeed this study reports an increased cardiovascular risk of T2D patients displaying HR values greater than 70 beats/minute as compared to those showing lower values [5].

Since HR is under parasympathetic but also sympathetic influences, one of the hypothesis advanced for explaining the adverse prognostic impact of elevated HR in T2D identifies the adrenergic activation as a potential pathophysiological mechanism responsible for this finding. This hypothesis is indeed based on the evidence that in number of diseases, characterized by a sympathetic activation [6] this neurogenic abnormality, which in T2D, however, not always is detectable [7], plays an adverse prognostic impact on cardiovascular morbidity and mortality independently on other confounders [8–10]. However, information on whether the sympathetic nervous system is differently activated in T2D patients displaying resting HR values above or below the above mentioned cutoff is at present lacking. Knowledge on this issue is critical for understanding whether more or less elevated HR values are capable to reflect a different degree of sympathetic activation in T2D. It is also useful in clinical practice as a simple tool for identifying T2D patients in which lifestyle and/or pharmacological interventions effective in modulating sympathetic cardiovascular drive are recommended.

In the present study we addressed the above mentioned issue by assessing in patients with T2D displaying different resting HR values two different independent neuroadrenergic markers, namely venous plasma norepinephrine (NE) and efferent postganglionic muscle sympathetic nerve traffic (MSNA), directly recorded via the microneurographic technique in the peroneal nerve [11]. The assessment of HR included not only clinic but also 24-h values, allowing to relate static and dynamic behaviour of this hemodynamic variable to the above mentioned sympathetic markers. To determine whether the relationships between HR and sympathetic variables were specific for T2D, we performed the same analysis in a group of healhy controls representative of the general population.

## Methods

### Study sample

The study population included 58 T2D patients with an age range between 39 and 57 years and 52 age-matched healthy controls. For both groups the analysis was performed retrospectively and it was based on the detection of an HR value below or above 70 beats/minute at the office visit performed the day preceding the microneurographic nerve traffic recording session made in the frame of different investigations carried out between 2017 and 2020 and in 2022 after the end of the COVID pandemia. All individuals included in the study were in sinus rhythm, and no subject had a history of myocardial infarction in the 12 months preceding the study or a clinical or laboratory evidence of valvular heart disease, congestive heart failure, thyroid dysfunction, hypertension, metabolic syndrome, obesity, renal failure or any other condition known to affect autonomic modulation of the cardiovascular system [6, 12]. Recruited DM patients were affected by T2D, with a reported duration amounting to 4.8 ± 1.8 (mean ± SEM) years. No patient was being treated or had symptoms of peripheral neuropathy. They were under oral antidiabetic drug treatment with metformin, sulphonylureas, dipeptidyl peptidase-4 inhibitors, alone or in combination and/or insulin and their metabolic control was satisfactory based on blood hemoglobinA1c and plasma glucose assessment. About 1/3 of the T2D patients were under lipid lowering treatment, while no patient was treated with antihypertensive drugs. Healthy controls were pharmacologically untreated. Both patients and controls were evaluated on an outpatient basis and gave their written consent to the study after being informed of its nature and purpose. The study sample size was determined taking into account that from our previuos studies the groups of subjects with low, medium and high values of HR display a mean MSNA value amounting to 40, 45 and 50 bursts/minute, respectively, with a 5 bursts/minute value of standard deviation [13, 14]. Calculations were made indicating that almost 10 patients per group are needed to have a power amounting to 90% with an alfa error of 5%. The study protocol was approved by the Ethics Committee of one of the institutions involved.

### Measurements

Measurements included body mass index, waist-to-hip ratio, sphygmomanometric and beat-to-beat finger systodiastolic blood pressure (BP) via a validated instrument (Ohmeda 2003; Finapres, Englewood, Florida, USA) [6–11], HR (EKG) and respiration rate (pneumotacograph). They also included MSNA via the microneurographic technique [6–14], venous plasma NE via high-performance liquid chromatography with electrochemical detection (Machery-Nagel ET 200/4 Nucleosil 100–5 C18 column, Machery-Nagel, and Waters 460 electrochemical detector; Waters GmbH, Eschborn, Germany) [15], and an echocardiographic assessment of left ventricular ejection fraction, measured from the four-chamber apical projection using the product area times length [16, 17]. Echocardiographic data also included left ventricular end-diastolic diameter and left ventricular mass index, calculated by the Devereux formulae and normalized to body surface area [17], and left atrial diameter determined at end-systole indexed to body surface area [18].

An EKG-Holter monitoring was performed during the 24-h period in the days preceding the evaluation of the sympathetic neural function. Simultaneous MSNA, beat-to-beat HR and BP recordings were digitized with a sampling frequency of 1000 Hz (PowerLab Recording System Model ML870 8/30; AD Instruments, Bella Vista, New South Wales, Australia). MSNA was quantified over a 30-min period as bursts incidence over time (bursts/minute) [8–11]. This quantification has been shown to be highly reproducible, that is to differ by only 4.3% when assessed on two separate occasions [19]. All measurements were performed in a single center following the same standardized protocol.

### Experimental design and statistical analysis

All participants were examined in the morning after a light breakfast and an overnight abstinence from alcohol and coffee consumption. They were asked to assume the lying position, after which three sphygmomanometric BP and HR (palpatory method, radial artery) were obtained. Following the BP and HR measurements, patients were fitted with an intravenous cannula and the devices to measure finger BP and to record an EKG. Blood samples were taken 30 min after positioning the venous cannula. A microelectrode was then inserted into a peroneal nerve to obtain MSNA, which was recorded together with finger BP and the EKG during a 30-min period. Data were collected in a semidark and quiet room kept at a constant temperature of 20–22 °C. As mentioned above, the study had a retrospective nature and included data collected and already blindly analyzed for other microneurographic studies not related to the present investigation. This allowed to avoid any potential bias in the data analysis. Values from individual participants were averaged (see below) and expressed as means ± SEM. The two study populations were subdivided into 3 different groups according to the clinic HR values. Comparisons between groups were made by one-way analysis of variance and chi-square test. Bonferroni correction was applied to compare two groups. The Pearson correlation coefficient was used to assess the relationships between HR, MSNA, and venous plasma NE, a *P* < 0.05 being taken as the minimal level of statistical significance. All statistical analyses were performed by SAS software version 9.4 (SAS Institute Inc, Cary, NC, USA).

## Results

### T2D patients group

As shown in Table 1 the 3 groups of T2D patients characterized by resting clinic HR values below 70, between 70 and 79 and ≥ 80 beats/minute displayed similar gender distribution and superimposable age. This was the case for body mass index, systolic and diastolic BP, echocardiographic parameters, plasma creatinine, plasma glucose and glicated hemoglobin. T2D patients belonging to the 3 HR groups did not significantly differ each other as far as the therapeutic scheme of the antidiabetic and lipid lowering drugs taken daily. There was, however, a non significant tendency in the group of patients with greater HR to include insulin more frequently in the treatment schedule (8 patients versus 6 patients in each of the other two groups groups of patients characterized by lower HR, *P* = NS).

**Table 1:** Demographic, anthropometric, hemodynamic and clinic characteristics of diabetic patients with different clinic heart rate (HR) values

Variable	HR < 70	HR 70–79	HR ≥ 80
	b/min (*n* = 21)	b/min (*n* = 20)	b/min (*n* = 17)
Age (yrs)	52.3 ± 2.1	51.8 ± 2.0	53.5 ± 2.2
Gender (M/F, n°)	16/5	16/4	14/3
BMI (kg/m^2^)	28.2 ± 0.9	27.8 ± 0.8	28.8 ± 1.0
Waist-to-hip ratio	0.91 ± 0.02	0.90 ± 0.02	0.92 ± 0.03
SBP (mmHg)	133.5 ± 2.1	134.2 ± 2.2	137.1 ± 2.4
DBP (mmHg)	78.5 ± 1.9	78.0 ± 1.7	77.5 ± 2.0
HR (beats/min)	65.2 ± 0.7	75.4 ± 0.6**	86.3 ± 1.1**^,††^
LVEF (%)	61.4 ± 1.3	62.6 ± 1.2	61.1 ± 1.4
LVEDD (mm)	50.1 ± 1.0	51.3 ± 1.1	49.8 ± 0.9
LVMI (g/m^2^)	102.8 ± 1.3	101.7 ± 1.3	103.3 ± 1.5
LAD indexed BSA (mm/m^2^) Plasma creatinine (mg/dl)	19.2 ± 0.2 0.98 ± 0.2	18.7 ± 0.2 0.99 ± 0.4	18.9 ± 0.3 0.95 ± 0.3
eGFR (ml/min/1.73 m^2^)	73.5 ± 6.1	74.3 ± .47	71.8 ± 6.6
Resp rate (breaths/min)	16.4 ± 1.7	16.3 ± 1.2	17.0 ± 1.5
Plasma glucose (mg/dl)	138.3 ± 6.9	140.5 ± 6.4	137.1 ± 7.7
Hb A1C (mmol/mol)	7.0 ± 0.2	6.9 ± 0.3	7.2 ± 0.2

Data are shown as means ± SEM. *M* male; *F* female, *BMI* body mass index, *SBP* systolic blood pressure, *DBP*: diastolic blood pressure, *LVEF* left ventricular ejection fraction, *LVEDD* left ventricular diastolic diameter, *LVMI* left ventricular mass index, *LAD* left atrial diameter, *BSA* body surface area, *eGFR* estimated glomerular filtration rate, *Resp rate* respiration rate, *HbA1c* glycated hemoglobin. ** *P* < 0.01 vs HR < 70 beats/minute_,_^††^ *P* < 0.01 vs HR 70–79 beats/minute

**Fig. 1:**
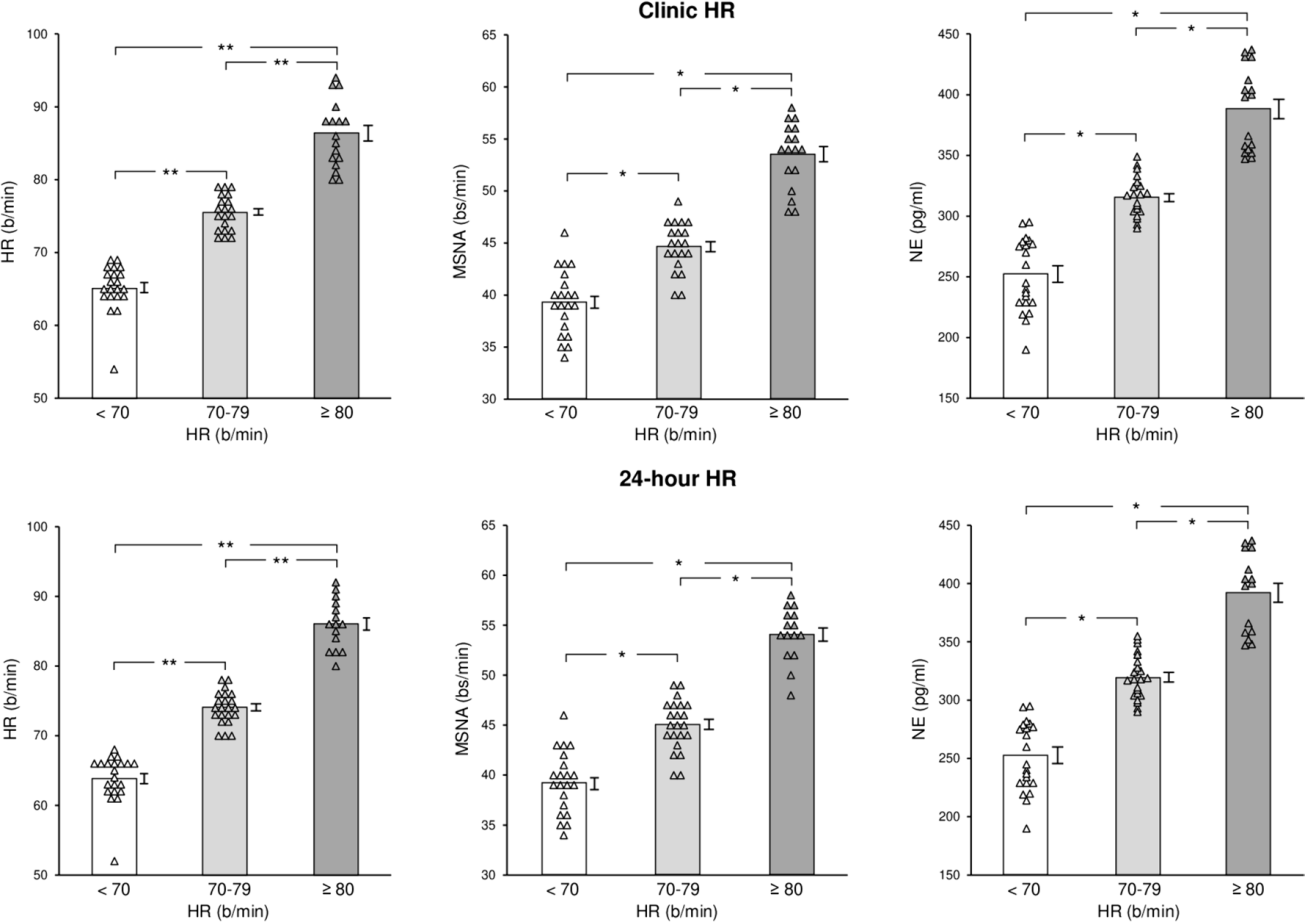
Individual and average values (± SEM) of heart rate (HR), muscle sympathetic nerve traffic (MSNA), and venous plasma norepinephrine (NE) in the groups of patients with type 2 diabetes mellitus (T2D) with clinic HR (upper panels) or 24-h HR (lower panels) values below 70 beats/minute, between 70 and 79 beats/minute and ≥ 80 beats/minute. Bs/min indicates bursts frequency over time (minute). *P < 0.01and **P < 0.001 refer to the statistical significance between groups

Individual and average clinic HR values assessed in the 3 groups are shown in Fig. 1, which also displays individual and average data related to 24-h HR Holter monitoring, MSNA and venous plasma NE. Resting HR values were progressively and significantly increased from the group characterized by HR values below 70 beats/minute to the ones displaying HR between 70 and 79, and ≥ 80 beats/minute (Fig. 1, left upper panel), respectively. This was the case also for 24-h HR values (Fig. 1, left lower panel). More importantly, although interindividual differences were detected, T2D patients with clinic HR values greater than 70 beats/minute were characterized by MSNA and plasma NE values significantly greater than those found in patients with HR below 70 beats/minute (Fig. 1, upper middle and right panels). The increase was progressively greater for magnitude from the group with HR between 70 and 79 beats/minute to the one with HR ≥ 80 beats/minute, respectively. A similar trend was detected in males and females and when HR values were assessed during the 24 h via Holter monitoring (Fig. 1, lower middle and right panels). T2D patients under insulin treatment showed a tendency, although not statistically significant, to display HR and MSNA values greater than those found in T2D not treated with insulin.

**Fig. 2:**
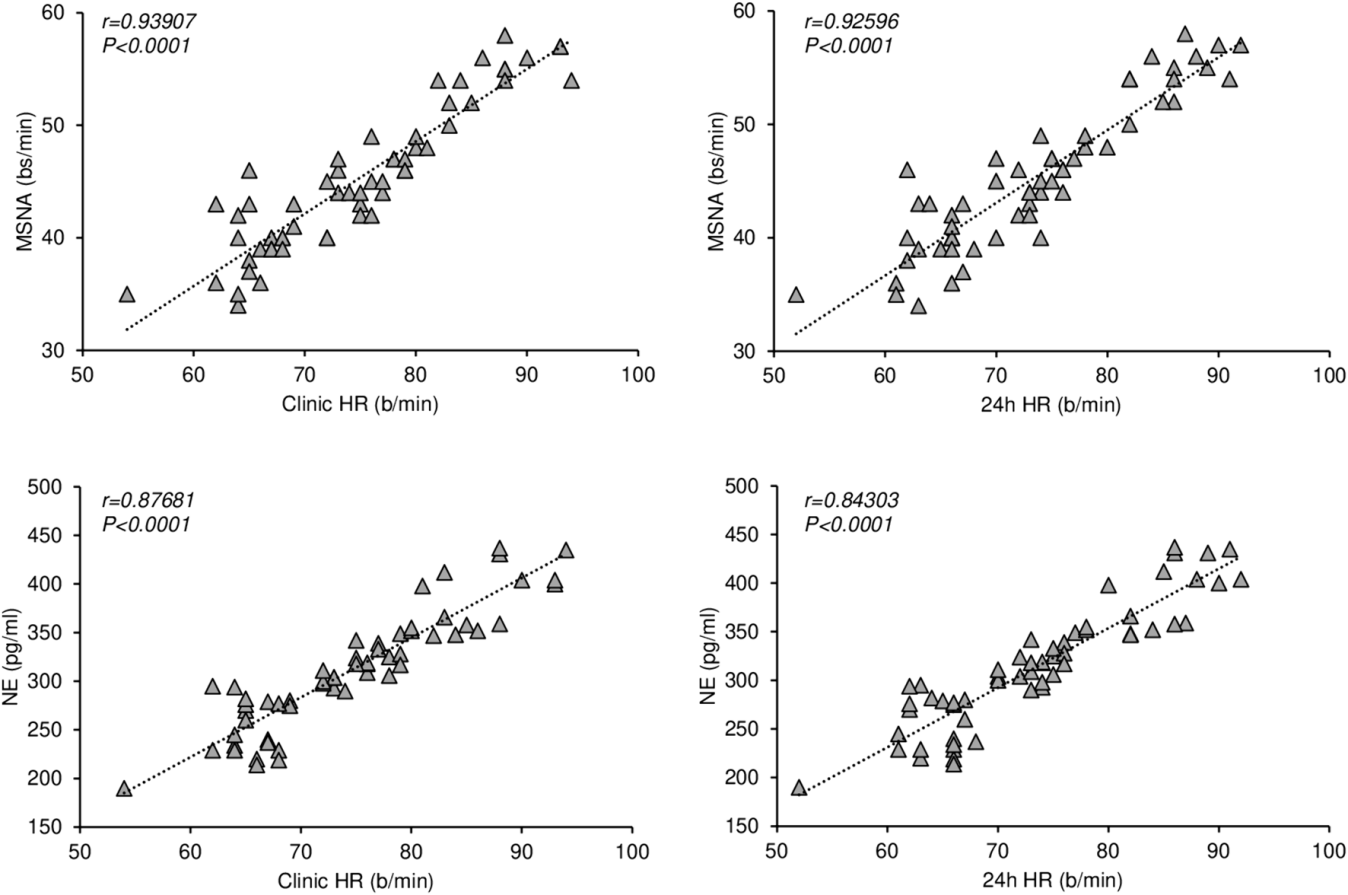
Left: Regressing clinic heart rate (HR) on muscle sympathetic nerve traffic (MSNA), expressed as bursts frequency over time (bursts per minute, bs/min, upper panel) and plasma norepinephrine (NE, lower panel) in 58 patients with T2D. Correlation coefficients (r) and *P* values are shown. Right: Regressing 24-h HR on MSNA, expressed as bursts frequency over time (bursts per minute, upper panel) and venous plasma NE (lower panel) in 58 patients with type 2 diabetes mellitus. Correlation coefficients (r) and *P* values are shown

As shown in Fig. 2, left upper and lower panels, in the T2D group MSNA and plasma NE showed a highly significant relationship with clinic HR, the correlation being closer for MSNA than for NE. Similar relationships were found when 24-h HR values were considered (Fig. 2, right upper and lower panels). When correlations were sought separately in the 3 different groups of T2D patients, MSNA was always significantly related to HR, while NE only in the group with HR values ≥ 80 beats/minute (data not shown).

### Healthy controls group

**Fig. 3:**
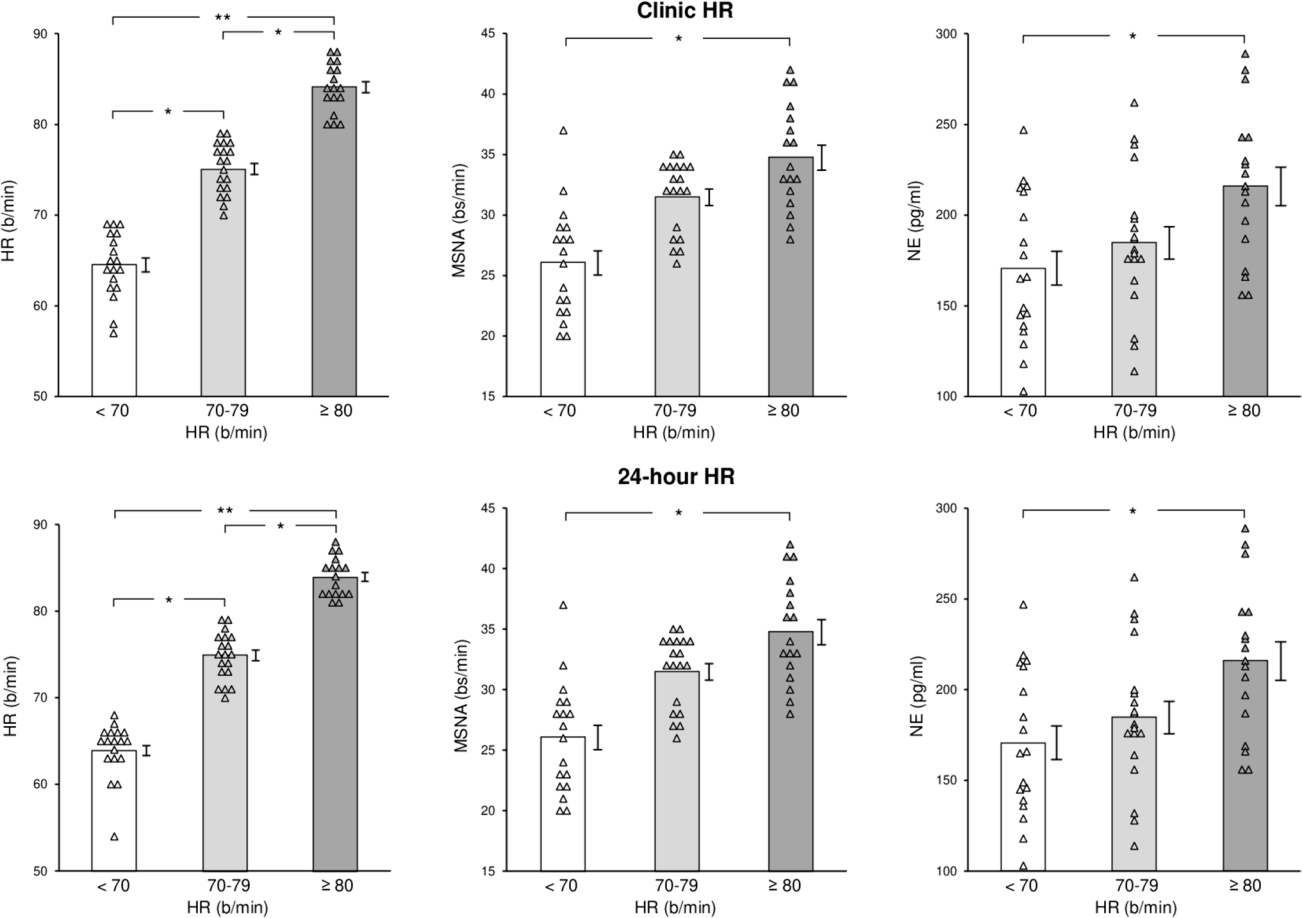
Individual and average values (± SEM) of heart rate (HR), muscle sympathetic nerve traffic (MSNA), and venous plasma norepinephrine (NE) in the groups of healthy subjects with clinic HR (upper panels) or 24-h HR (lower panels) values below 70 beats/minute, between 70 and 79 beats/minute and ≥ 80 beats/minute. Bs/min indicates bursts frequency over time (minute). *P < 0.01 and **P < 0.001 refer to the statistical significance between groups

As shown in Table 2, the 3 groups of healthy control subjects displaying different resting clinic HR values, i.e., below 70, between 70 and 79 and ≥ 80 beats/minute, showed similar values of the various anthropometric, hemodynamic, echocardiographic and biochemical variables examined. The corresponding individual and average clinic HR, 24-h HR, MSNA and plasma NE values are shown in Fig. 3. Resting HR values were progressively and significantly increased from the group with an HR below 70 beats/minute to the ones displaying HR between 70 and 79, and ≥ 80 beats/minute (Fig. 3, left upper panel). This was the case also for 24-h HR values (Fig. 3, left lower panel). Differently from what reported above in T2D patients, only healthy controls showing clinic HR values > 80 beats/minute displayed MSNA and plasma NE values significantly greater than those found in controls with HR below 70 beats/minute (Fig. 3, upper middle and right panels). Furthermore, at variance from what reported in T2D, in the group of healthy controls neither MSNA nor NE values were significantly related to clinic or 24-h HR (MSNA: *r* = 0.22 and *r* = 0.25, respectively, *P* = NS for both; NE: *r* = 0.19 and *r* = 0.21, respectively, *P* = NS for both). No statistically significant differences were found in the anthropometric indices and in the diastolic blood pressure values between T2D patients and controls. Systolic blood pressure, although remaining in the normotensive range, was significantly higher in TD2 patients than in controls.

**Table 2:** Demographic, anthropometric, hemodynamic and clinic characteristics of healthy control subjects with different clinic heart rate (HR) values

Variable	HR < 70	HR 70–79	HR ≥ 80
	b/min (*n* = 18)	b/min (*n* = 17)	b/min (*n* = 17)
Age (yrs)	48.7 ± 2.8	50.0 ± 2.9	50.6 ± 2.8
Gender (M/F, n°)	14/4	13/4	15/2
BMI (kg/m^2^)	26.7 ± 1.1	26.1 ± 1.3	26.5 ± 1.2
Waist-to-hip ratio	0.87 ± 0.03	0.86 ± 0.04	0.87 ± 0.03
SBP (mmHg)	126.2 ± 2.5	124.9 ± 2.2	127.9 ± 2.7
DBP (mmHg)	76.6 ± 1.8	77.1 ± 1.5	77.0 ± 2.3
HR (beats/min)	64.5 ± 1.1	73.3 ± 0.9**	84.8 ± 1.3**^,††^
LVEF (%)	63.8 ± 1.5	65.2 ± 1.7	64.1 ± 1.5
LVEDD (mm)	51.0 ± 1.2	51.9 ± 1.3	51.6 ± 1.3
LVMI (g/m^2^)	100.5 ± 1.4	98.9 ± 1.4	102.3 ± 1.6
LAD indexed BSA (mm/m^2^)	19.6 ± 0.2	19.9 ± 0.3	19.1 ± 0.2
Creatinine (mg/dl)	0.90 ± 0.4	0.93 ± 0.6	0.88 ± 0.4
eGFR (ml/min/1.73 m^2^)	76.6 ± 8.1	78.3 ± 9.8	78.8 ± 8.9
Resp rate (breaths/min)	15.3 ± 1.5	14.8 ± 1.3	15.9 ± 1.4
Plasma glucose (mg/dl)	87.8 ± 5.9	92.4 ± 8.8	89.3 ± 7.1

Data are shown as means ± SEM. *M* male; *F* female, *BMI* body mass index, *SBP* systolic blood pressure, *DBP* diastolic blood pressure, *LVEF* left ventricular ejection fraction; *LVEDD* left ventricular diastolic diameter, *LVMI* left ventricular mass index, *LAD* left atrial diameter, *eGFR* estimated glomerular filtration rate, *Resp rate* respiration rate. ** *P* < 0.01 vs HR < 70 beats/minute_,_^††^ *P* < 0.01 vs HR 70–79 beats/minute

## Discussion

Our study was aimed at assessing whether and to what extent increased HR values measured at rest in the clinical setting are capable to detect different degrees of sympathetic activation in T2D patients. It was also designed at defining a possible HR treshold value reflecting in clinical practice the presence in T2D patients of an augmented sympathetic cardiovascular drive. It was finally designed at determining whether the relationships between HR and sympathetic markers found in T2D were specific for this disease or rather also common to the healthy state. The study results, which provide a number of new information on the above mentioned issues and on other related aspects, can be summarized as follows. First, in patients with T2D without any evidence of autonomic neuropathy clinic HR values above 70 beats/minute are accompanied by a sympathetic activation significantly and markedly greater in magnitude than the one detected in patients in which T2D is associated with clinic HR values below 70 beats/minute. This finding therefore suggests that this HR cutoff value, which has been employed in different clinical trials for defining an increased cardiovascular risk [3–5], allows to detect diabetic patients with a lower or a higher neuroadrenergic drive to the heart and the peripheral circulation as well. Second, this treshold value is reliable not only when HR is measured in the clinical setting via the palpatory method but also when HR is dynamically evaluated via the Holter monitoring throughout the 24 h period. Third, the results are almost superimposable when sympathetic activity was directly quantified via the microneurographic technique as MSNA and when neuroadrenergic drive was assessed via a less direct approach based on the assay of venous plasma NE. Fourth, the study findings provide evidence that the assessment of the adrenergic cardiovascular drive was, nevertheless, less sensitive when based on NE assay than on MSNA recording, this being particularly the case when subanalyses of patients subgroups classifed according to less or more elevated HR values were performed. Finally, the relationships seen in T2D between HR and sympathetic markers appear to be specific for T2D patients, being not detectable in the healthy control individuals and unreated to the concomitant presence of an obese or an hypertensive state.

Establishing cutoff values of clinical HR values capable to reflect a greater sympathetic activation carries relevant pathophysiological and clinical implications, considering that an augmented sympathetic cardiovascular drive is associated in a number of cardiovascular disease, such as in the acute post-stroke phase, in acute myocardial infarction, in chronic kidney disease and chronic heart failure, with an elevated risk of fatal and non-fatal cardiovascular events [20–23]. This is the case also in T2D, in which the detection of a sympathetic overdrive, via assessment of direct and indirect markers of neuroadrenergic function, is associated, even before the development of an overt autonomic neuropathy, with an increased risk of cardiovascular, cerebral and renal complications as well as with a reduced survival rate [24–26]. It should be emphasized that this is the case even when data are adjusted for confounders, strengthening the prognostic relevance of the sympathetic alterations. In addition, a marked sympathetic activation may carry in diabetes an unfavorable impact on glycemic profile and insulin resistance [27].

Our study was not designed at determining the mechanisms responsible for the sympathetic activation characterizing T2D. A number of factors may be responsible for this alteration, including the metabolic abnormalities occurring in T2D [8, 10, 27]. Based on the data collected in previous studies [8, 9] we can speculate that this neurogenic abnormality depends to a greater extent on an alteration of the reflex control of vagal and sympathetic cardiovascular influences physiologically exerted by the arterial baroreceptors. According to data collected by our group and others, the baroreflex-sympathetic alteration appears to be more pronounced in T2D as compared to what found in other diseases, such as for example esssential hypertension [6, 8, 10].

An additional result of our study deserves to be briefly mentioned. This refers to the question as to whether the HR treshold for the sympathetic activation detected in T2D is specific for this disease or rather it can be generalized to the healthy state and/or to other pathological conditions also characterized by sympathetic abnormalities. As far as healthy subects are concerned, our data show that HR cutoff for identifyng a significant increase in the different sympathetic markers can be located at higher HR values, namely ≥ 80 beats/minute. On the other hand, as far as patients is concerned, while the same HR cutoff value defined in our T2D patients was detected also in chronic heart failure, a higher one was found in essential hypertension [13, 28]. This may suggest that no extrapolation of the present data to the healthy state or to other diseases with evidence of sympathetic activation can be made. Similarly, no extrapolation of the present data collected in patients with T2D can be made to those with T1D, which are not characterized by an increased sympathetic cardiovascular drive [8–10].

### Limitations and clinical implications

Our study has strengths and limitations. The strengths include (1) the multiple assessment of sympathetic cardiovascular drive, which was based on two independent neuroadrenergic markers, namely venous plasma NE and MSNA, and (2) the inclusion in the study population of healthy control subjects, allowing us to determine the specificity of the relationships between HR and the sympathetic markers seen in T2D patients. The limitations include the retrospective nature of our study and the relatively small number of T2D patients evaluated. The latter limitation can be ascribed to the strict criteria of inclusion of T2D patients we adopted in our study as well as to the technical difficulties to obtain optimal microneurographic MSNA recordings in humans. A further limitation is represented by the fact that we examined treated T2D patients, since ethical and clinical considerations prevented antidiabetic drug treatment to be withdrawn in our study population. It should be noted, however, that (1) the quite homogeneous distribution of antidiabetic and lipid lowering drugs in the 3 groups of patients with different HR should rule out the possibility that drug treatment may have affected the results and (2) the different drugs on which the antidiabetic therapeutic intervention was based display, if anything, modest sympathomodulatory effects [29]. These may counterbalance, together with statins [30], the sympathoexcitatory effects triggered by chronic insulin administration [31], which are markedly attenuated in the chronic insulin resistant states compared to those seen experimentally during acute insulin administration [32, 33].

Considering the adverse clinical impact of the sympathetic overdrive on patients’ prognosis, the results of the present study underline the potential relevance of lifestyle measures and pharmacological interventions counteracting the reported neuroadrenergic abnormality [34]. As far as drug treatment is concerned, compounds with sympathomoulatory properties on the heart with a resulting HR reduction should be indicated, such as central sympatholytics, ibravadine and beta_3_ adrenoreceptors antagonists, which, at variance from classic beta blocking drugs, are almost devoid of any adverse effect on the glycemic profile [34].

## Conclusions

In summary, in T2D patients clinic HR values greater than 70 beats/minute allow to detect individuals in which sympathetic cardiovascular influences are more markedly activated and, as mentioned above, may have greater benefits from neuromodulatory sympathomoderating therapeutic interventions.

## Data Availability

The data that support the findings of this study are not openly available due to reasons of sensitivity and are available from the corresponding author upon reasonable request.
